# Artificial Cutaneous Sensing of Object Slippage using Soft Robotics with Closed‐Loop Feedback Process

**DOI:** 10.1002/smsc.202100002

**Published:** 2021-02-07

**Authors:** Tomohito Sekine, Yi-Fei Wang, Jinseo Hong, Yasunori Takeda, Reo Miura, Yushi Watanabe, Mai Abe, Yoshiki Mori, Zhongkui Wang, Daisuke Kumaki, Fabrice Domingues Dos Santos, Atsushi Miyabo, Sadao Kawamura, Shizuo Tokito

**Affiliations:** ^1^ Research Center for Organic Electronics (ROEL) Graduate School of Science and Engineering Yamagata University 3-4-16, Jonan Yonezawa Yamagata 992-8510 Japan; ^2^ Department of Robotics Ritsumeikan University 1-1-1 Noji-higashi Kusatsu Shiga 525-8577 Japan; ^3^ Piezotech S. A. S. Arkema-CRRA Rue Henri Moissan Pierre-Benite Cedex 63493 France; ^4^ Arkema Kyoto Technical Center 93, Chudoji, Awatacho Shimogyo-ku Kyoto 600-8815 Japan

**Keywords:** cutaneous sensing, robotic grippers, soft robotics, soft sensors, tactile feedback system

## Abstract

Tactile sensing is desirable for skillful object handling in soft robotics applications. Real‐time measurement and identification of dynamic shear forces are crucial for slip detection and object interaction. This study proposes a soft sensor with a closed‐loop feedback system for dynamic shear force detection to address object slippage in a soft robotic gripper. The sensor is made of a ferroelectric polymer with nanocarbon materials because of the resulting improved crystallinity and good sensitivity. The sensor shows high performance and high‐speed response for detecting dynamic shear forces when fragile objects (e.g., vegetables) slip from the soft gripper. The artificial cutaneous sensor shows high sensitivity for grasping such objects with the gripper. Furthermore, the feedback system provides a control system for operations and avoids the need for training for various tasks, thus demonstrating the potential of the proposed system for novel soft robotics applications such as biomimetic electronic skin.

## Graphical abstract

Tactile sensing is desirable for skillful object handling in soft robotics applications. A soft sensor with a closed‐loop feedback system is proposed for shear force detection to address object slippage in a soft robotic gripper. Furthermore, the feedback system provides a control system for operations and avoids the need for training for various tasks.

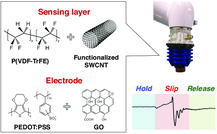

## 1 Introduction

Multiaxial industrial robots have facilitated innovative component assembly in the fields of automotive and electronic device manufacturing^[^ ^1^, ^2^ ^]^ over the last decade.^[^ ^3^ ^]^ Some common problems with these conventional industrial robots include the fact that the equation of motion (EOM) for special coordinates needs to be solved to enable these robots to grasp several objects accurately and quickly^[^ ^4^ ^]^ and that such robots cannot deal with unexpected events (e.g., dropping or colliding with objects) because of the lack of tactile sensors. To solve these problems, soft robots with functional soft sensors have attracted considerable research attention in recent years.^[^ ^5^ ^]^ Functionalized flexible sensors that enable tactile sensing,^[^ ^6^ ^]^ touch sensing,^[^ ^7^ ^]^ optical sensing,^[^ ^8^ ^]^ and vision sensing^[^ ^9^ ^]^ on robots have been demonstrated to reproduce the human cutaneous sense, which makes them potentially useful in soft robotics applications.^[^ ^4^, ^10^, ^11^ ^]^ Moreover, robots with such sensors can be used for applications in artificial intelligence (AI) and big data,^[^ ^12^ ^]^ and therefore, they have now become usable in tasks such as sorting foods.

Soft robots are expected to find various industrial applications. To realize accurate control using soft robots, it is important to understand object behaviors once they are grasped. The introduction of soft tactile sensors for soft robots is in its infancy and there are still many issues. Especially, existing inorganic rigid sensors cannot be used in this regard because they are not sufficiently soft. At the same time, studies have recently developed soft sensors that are sensitive to various stimuli such as axial compression,^[^ ^13^, ^14^ ^]^ temperature,^[^ ^15^, ^16^ ^]^ stretching strain,^[^ ^17^, ^18^ ^]^ and bending strain.^[^ ^19^, ^20^ ^]^ Existing platforms such as piezoresistive‐ or capacitive‐based array sensors can measure static strain changes in soft robots.^[^ ^21^, ^22^, ^23^ ^]^ Moreover, Ntagios and Dahiya et al. recently reported description of 3D‐printed capacitive sensors on soft materials of graphite ink.^[^ ^24^ ^]^ In addition, fingerprint‐enhanced capacitive flexible sensor was also reported for discrimination of static and dynamic tactile signals.^[^ ^25^ ^]^ In these works, the authors suggested human skin inspired functional flexible sensors based on capacitive structure for tactile signal detection with robot systems.

In particular, shear force sensing has attracted attention because it plays an important role in haptic controls.^[^ ^26^, ^27^ ^]^ Therefore, the dynamic shear force still needs to be evaluated. Furthermore, acquired slipping signals depend on the shearing behavior, which depends on the object shape and its identified surface state. Thus, improved identification can help improve the sensing abilities of soft robotics. By contrast, slippage sensing is fundamentally difficult in soft robotics. Piezoelectric soft sensors made of ferroelectric materials show high performance and high‐speed response for detection of applied stress and vibrations against objects slippage. Moreover, it does not require a power supply and array construction to detect the signals as compared with piezoresistive or capacitive sensors.^[^ ^28^, ^29^ ^]^ Although piezoelectric sensors are useful for dynamic signal detection, it remains difficult to realize a high‐sensitivity having a stable signal as fabricating soft sensor. Recently, some studies have proposed using ferroelectric materials to produce sensors for rigid and soft robots;^[^ ^30^, ^31^ ^]^ they have also suggested the benefits of using piezoelectric materials for robotic sensors. However, no studies have been reported on shear force sensing for soft robots because the organization of the required material systems, including the required sensor characterization, is essentially a difficult task. It is necessary to maintain or improve the sensitivity for shear force detection using ferroelectric materials for soft sensing. Therefore, it remains challenging to exploit e‐skin capabilities for soft robotics and to develop a complex soft sensory system with high‐performance soft sensors made of ferroelectric materials.

In this article, we fabricated a highly sensitive soft sensor composed of a ferroelectric polymer and nanocarbon materials to measure the dynamic shear force with a high‐speed response. A printing process is superior to other approaches because it enables combining several functional materials to fabricate a layer‐by‐layer device. The sensing ability of our sensor was increased substantially by using nanocarbons and controlling the annealing processes to rearrange the crystallinity of the sensing layer. Consequently, it was possible to employ ferroelectricity beyond 11.0 μC cm^−2^ and to detect a high acceleration value of 4.0 dV ds^−1^ with an applied force speed of 200 mm s^−1^. This ferroelectricity is approximately two times that reported in previous studies.^[^ ^32^ ^]^ Moreover, our sensor can be mounted on a soft robotics gripper with a co‐robot, and it can control a robotic motion with a closed‐loop feedback process. This enables the robot to regrip objects, such as fragile glass bottles and vegetables, when they start to slip. This study offers a general platform for next‐generation e‐skin for soft robot sensing applications.

### 1.1 Material System for Soft Sensors

**Figure 1:**
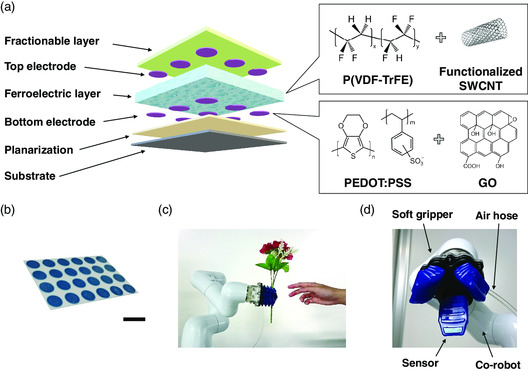
Fabrication of soft sensor for tactile sensing with co‐robot. a) Schematic of device fabrication of soft sensor using the functional polymers P(VDF–TrFE) and PEDOT:PSS and nanocarbon materials. The sensor consists of five layers: planarization, bottom electrode, ferroelectric layer, top electrode, and fractionable layer. b) Photograph of sensor. Scale bar: 10 mm. c) Co‐robot and a soft gripper equipped with the soft sensor. d) Close‐up view of robot system consisting of co‐robot, soft gripper, air hose, and soft sensor.

The soft sensor is fabricated from a ferroelectric polymer ideal for shear force sensing due to its high‐speed response.^[^ ^33^ ^]^ We used poly(vinylidene fluoride‐co‐trifluoroethylene) [P(VDF–TrFE)] as a sensing layer. In our study, we combined the VDF–TrFE polymer with nanocarbon material of a single‐wall carbon nanotube (SWCNT) to fabricate the sensor. The crystallinity of the VDF polymer can be orientated by mixing it with SWCNT because of the rearrangement of the polymeric lamella crystal.^[^ ^34^ ^]^ To realize the aforementioned materials system, we mixed a polar solvent of *N*‐methylpyrrolidone (NMP) with P(VDF–TrFE) and SWCNT. Moreover, poly(3,4‐ethylenedioxythiophene):poly(4‐styrenesulfonate) (PEDOT:PSS) was mixed with a graphene oxide (GO) to fabricate the electrode of the sensor. The GO also can improve the crystallinity of P(VDF–TrFE).^[^ ^35^ ^]^ Interactions between the fluorine groups of P(VDF–TrFE) and carbonyl functional groups of GO in PEDOT:PSS assist polymer chain rotation for the formation of the lamella crystal in the interface of both layers.^[^ ^36^ ^]^ Figure S1, Supporting Information, shows details of the printing and fabrication processes, and Figure S2, Supporting Information, shows the characteristics of the electrode. The solutions for both the electrode and the ferroelectric layer were formed onto a plastic substrate (**Figure** **1**). To control object slippage, a polyimide (PI) layer with low dynamic friction coefficient was affixed on our sensor (Figure 1a). Figure S3, Supporting Information, shows the frictional coefficient of the PI film, and Figure 1b shows a photograph of our soft sensor. The printing process can produce a considerably high surface flatness for various layers.

In general, a polarization *P* is generated by the application of a strain *X* to ferroelectric polymeric crystals as follows^[^ ^37^ ^]^

(1) $$P_{i}=\sum_{j,k=1}^{3}d_{ijk}X_{jk}$$

where *d* is the piezoelectric constant; it is a third‐rank tensor. Consequently, ferroelectric materials show a piezoelectric effect. The sensor produces the following voltage signal under applied strains^[^ ^38^, ^39^ ^]^

(2) $$V=\rho A(d_{31}\frac{d\sigma_{1}}{dt}+d_{32}\frac{d\sigma_{2}}{dt}+d_{33}\frac{d\sigma_{3}}{dt})$$

where *V* is the generated voltage; *ρ* is the impedance of the sensor and the measurement circuit; *A* is the overlapping area between the two electrodes; *σ*_1_, *σ*_2_, and *σ*_3_ are applied stresses in three dimensions; *t* is the time; and *d*_31_, *d*_32_, and *d*_33_ are the piezoelectric constants of the ferroelectric layer. Equation (1) indicates both the directions and values of the stresses. By using Equation (1), our sensor can detect signals corresponding to short‐range object slippage from the hands. When this sensor is implemented on the gripper, it enables the robots to precisely grasp various objects with synchronization through a driving program. Figure 1d shows the collaboration between a human and a robot in a pick‐and‐place activity; Figure 1e shows a photograph of the sensor mounted on the gripper. This pneumatic gripper made of dimethylpolysiloxane can withstand a pressure of up to 100 kPa.

## 2 Results

### 2.1 Tactile Soft Sensor Fabrication and Characterization

**Figure 2:**
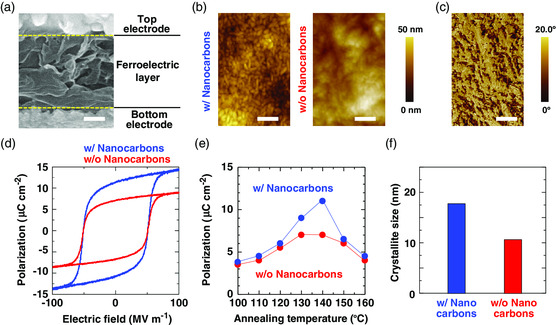
Electric characteristics of soft sensor. a) Cross‐sectional SEM image of sensor. Scale bar: 1 μm. b) Surface topographic images of ferroelectric layer with and without nanocarbon materials after annealing as obtained using AFM spectroscopy. Scale bars: 100 nm. c) Surface phase image of ferroelectric layer with nanocarbon materials after annealing. Scale bars: 1 μm. d) Polarization–electric field (*P*–*E*) hysteresis loops of soft sensor. e) Polarization of the sensor as a function of annealing temperature. f) Crystallite size of ferroelectric layer with and without nanocarbon materials.

**Figure** **2** shows the characteristics of the soft sensor and the ferroelectric layer in addition to the morphological behavior after thermal annealing. Figure 2a shows a cross‐sectional scanning electron microscopy (SEM) image of the sensor. The sensor comprises of two electrodes and a 2 μm thick ferroelectric layer consisting of P(VDF–TrFE) and nanocarbon materials. Figure 2b,c, respectively, show the surface topography and phase images of the ferroelectric layer as obtained using atomic force microscopy (AFM). In the topography information, the root mean square (RMS) surface roughness was 10.0 and 15.0 nm. Moreover, the SWCNTs were distributed over the P(VDF–TrFE) layer due to the existence of functional groups. In fact, the phase image did not indicate aggregation points (Figure 2c). The phase image indicates an adsorption and viscoelasticity of sample surface; therefore, P(VDF–TrFE) can form uniform ferroelectric layers on the electrode. These results show that nanocarbon materials did not affect the morphological behavior of the surface state. Figure 2d shows the polarization–electric field (*P*–*E*) hysteresis loop of our sensor with and without nanocarbon materials. In this article, we define the Pr value as the intersection of the polarization and the electric field curves with the zero of the coordinate systems. The larger the hysteresis window of our soft ferroelectric sensor, the higher is its performance. Surprisingly, the loop with nanocarbon materials realized ferroelectricity of over 11.0 μC cm^−2^ despite the use of the screen‐printing process (current is ≈7.0 μC cm^−2^). These results were first demonstrated using solution technologies.^[^ ^29^, ^40^, ^41^, ^42^ ^]^ These good polarization values indicate that our sensor can show high sensitivity to the applied strain. The aforementioned value enables high‐sensitivity detection of the shear force. Figure 2e shows the sensor's Pr values as a function of the annealing temperature. The highest Pr values were obtained for an annealing temperature of 140 °C. These results indicate that nanocarbon materials promoted the growth of the polymeric crystallinity of the sensing layer only in the mixing processes. In fact, X‐ray diffractometry (XRD) calculations of the crystalline sizes of P(VDF–TrFE) revealed that the nanocarbon materials influenced the growth of the polymeric crystal. Figure S4, Supporting Information, shows the basal XRD spectra for the calculated crystalline sizes. The crystallinity with nanocomposites may initially increase because of the interaction between the surface charges via delocalized *π*‐electrons with the remaining functional groups of SWCNT and VDF–TrFE polymers. These interactions lead to a gradual increase in the nucleation rate of the polar β‐phase.^[^ ^34^, ^35^, ^36^ ^]^

### 2.2 Sensing Performance for Dynamic Shear Force

**Figure 3:**
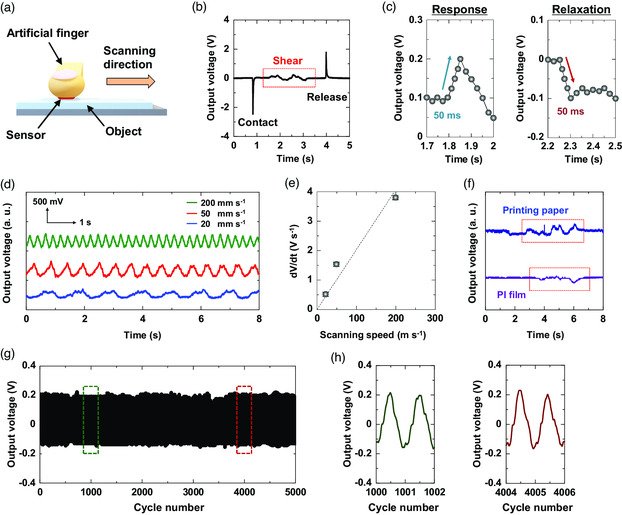
Response characteristics of soft sensor for applied shear force. a) Schematic 3D illustration of shear force application for sensor with artificial finger. Sensor was attached on the finger using a PI film. The object was glass. b) Sensor response for shear force application. When artificial finger made contact with and released the object, a voltage spike was generated because our sensor produces a differential calculus signal. Upon applying the shear force, a periodic signal was generated. c) Measured response and reaction signals enlarged from Figure 3b. d) Dependence of signal frequency on speed of applied shear force. Speeds of 20, 50, and 200 mm s^−1^ were tested. e) Detected accretion behavior as a function of scanning speed. Accretion was calculated by dividing the generated voltage by time. The average values for three samples were plotted. f) Change in signals with surface material of objects. g) Cycling test illustrating stability of shear response over 5000 cycles. No signal drift was measured after 5000 cycles. h) Representative signal data for 1000 and 4000 cycles.

**Figure** **3** shows the sensing response of the soft sensor for an applied shear force. Figure 3a shows a schematic 3D illustration of the measurement setup for characterizing the sensing ability. First, we attached the sensor to an artificial finger using PI film. The scanning speed was 20 mm s^−1^. We applied an axial compression force of 1.0 n through the artificial finger on the object. In this article, we calculated the shear forces by a synthetic vector in the perpendicular and parallel directions. The axial compression force is not time varying. By contrast, the shear forces (force of scanning direction) depended on the scanning speeds and only the changes in the parallel force. Figure S5, Supporting Information, shows details of the measurement setup for shear force application. Figure 3b shows the sensor response upon applying a shear force. When the artificial finger came in contact with and released the object, a voltage spike was generated because our sensor produced a piezoelectrical signal. When we applied the shear force, a periodic signal was generated. The mean magnitude and frequency of the signal depend on the applied force. In Figure S6, Supporting Information, experimental result to quantify the shear force sensing capability is shown. Figure 3c shows an enlarged view of the measured responses and reaction signals from Figure 3b; both response times were under 50 ms. Figure 3d shows the variation of the signal frequency for three applied speeds: 20, 50, and 200 mm s^−1^. Generally, the output voltage and their signal frequency in the piezo devices depend on the scanning speeds because the signals are evaluated by a differential equation for the applied stress (given by Equation (2)). When the speeds change, the evaluated values also change. These results indicate that the signal frequency increased with the speed of the applied force. Figure 3e shows the behavior of the calculated accretion values as a function of the scanning speed. The accretion value was calculated by dividing the generated voltage shown in Figure 3d by the time. This result indicates that our soft sensor can be used to monitor various slippage conditions. The scanning speeds affected the response time of the sensor. The sensor shows extremely high trackability for the applied shear force, as indicated by the agreement between the generated signal frequency and the applied force. The high linearity indicates that the sensor can follow the object motion exactly. The signal strength and frequency of the sensor change depending on the surface state and material of the objects, as shown for the examples of general printing paper and PI film in Figure 3f. The surface roughness of the objects also affected the signal frequency.^[^ ^40^ ^]^ Material changes also impact the output voltage because the surface treatment changes. Furthermore, the output voltage depended on the frequency of the surface roughness of the objects. Figure 3g,h show cycling test results illustrating the stability of the shear response over 5000 cycles. The scanning speed was 20 mm s^−1^. Figure 3h shows representative signal data for 1000 and 4000 cycles. No signal drift was seen after 5000 cycles. These results demonstrate that the proposed high‐sensitivity soft sensor shows a high‐speed response for measuring applied shear forces.

### 2.3 Organization of Tactile Feedback System using Soft Sensor

**Figure 4:**
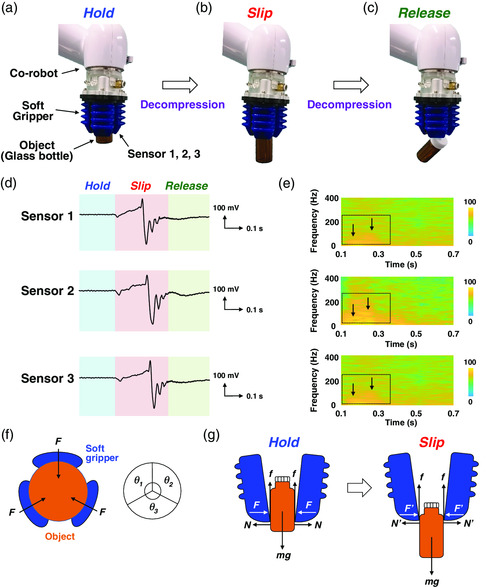
Object gripping using the three‐fingered robot with soft sensors. a–c) Photographs of sensing system with co‐robot, soft gripper, and soft sensors, respectively. The robot gripper can grasp objects through air compression. After decompression, the object is released and therefore slips. d) Shear force signals detected using sensors. The detected signals indicate hold, slip, and release states during object slipping. e) S–S spectra of piezoelectric voltage signals for several frequency ranges: (top) 100 and 180 Hz, (middle) 80 and 180 Hz, and (bottom) 100 and 180 Hz. Color bars indicate the intensity of frequency. Black arrows indicate the higher frequency in each calculated S–S signal. f) Object grasping model using three‐fingered gripper with soft sensors that shows force balance between the object and the fingers. *θ* is the angle at which force is applied on the object. g) Cross‐sectional model describing S–S mechanism during object grasping with three‐fingered gripper with soft sensors.

**Figure** **4** shows how robotic sensing abilities were tested experimentally. The soft robot gripper can detect tactile information without loss of softness by using our soft sensor. Three of our proposed soft sensors were attached on the gripper to measure the shear information of objects in real time, and a glass bottle was used as the object to be grasped. Figure 4a–c show photographs of our sensing system. The gripper holds the object with an inflation pressure of 30 kPa by air. We calculated the shear forces in the slippage direction by using a synthetic vector in the perpendicular and parallel directions (Figure S7, Supporting Information). First, the gripper with our sensor grasped the glass substrate with an inflation pressure of 30 kPa (Figure 4a). Next, the object slipped due to decompression (Figure 4b). Finally, the object was completely released by the gripper (Figure 4c). Figure 4d shows the shear force signals detected using the sensor when the object slipped. The signals obtained from all three sensors had similar shapes when the object slipped; the signals consisted of hold, slip, and release phases. In the slip phase, periodic stick–slip (S–S) signals were generated due to the spontaneous jerking motion between the two slipping objects. Moreover, the spectra repeat periodically as long as the object falls at a constant velocity. The S–S spectra of the voltage features exhibit time‐dependent variations, as shown in Figure 4e. We found that the glass bottle with a low frictional coefficient produces S–S signals with a frequency of ≈200 Hz. Figure 4f shows the object grasping model with the three‐fingered soft gripper; when the three fingers grasp the object, they all produce an equal holding force (*F*) on the object, with each force being applied at an angle of 120°. The cross‐sectional model in Figure 4g describes the S–S mechanism during grasping with the three‐fingered gripper; here, *F* and *N* are, respectively, the applied and repulsive forces, and *m*, *g*, and *f* are, respectively, the object mass, gravitational acceleration, and force in the EOM. In this scenario, several forces are balanced; specifically, *F* = *N* and *f* = *mg*. After decompression because the forces become imbalanced, the object slips; specifically, *F* > *F*′ and *mg* > *f*.^[^ ^43^, ^44^ ^]^

**Figure 5:**
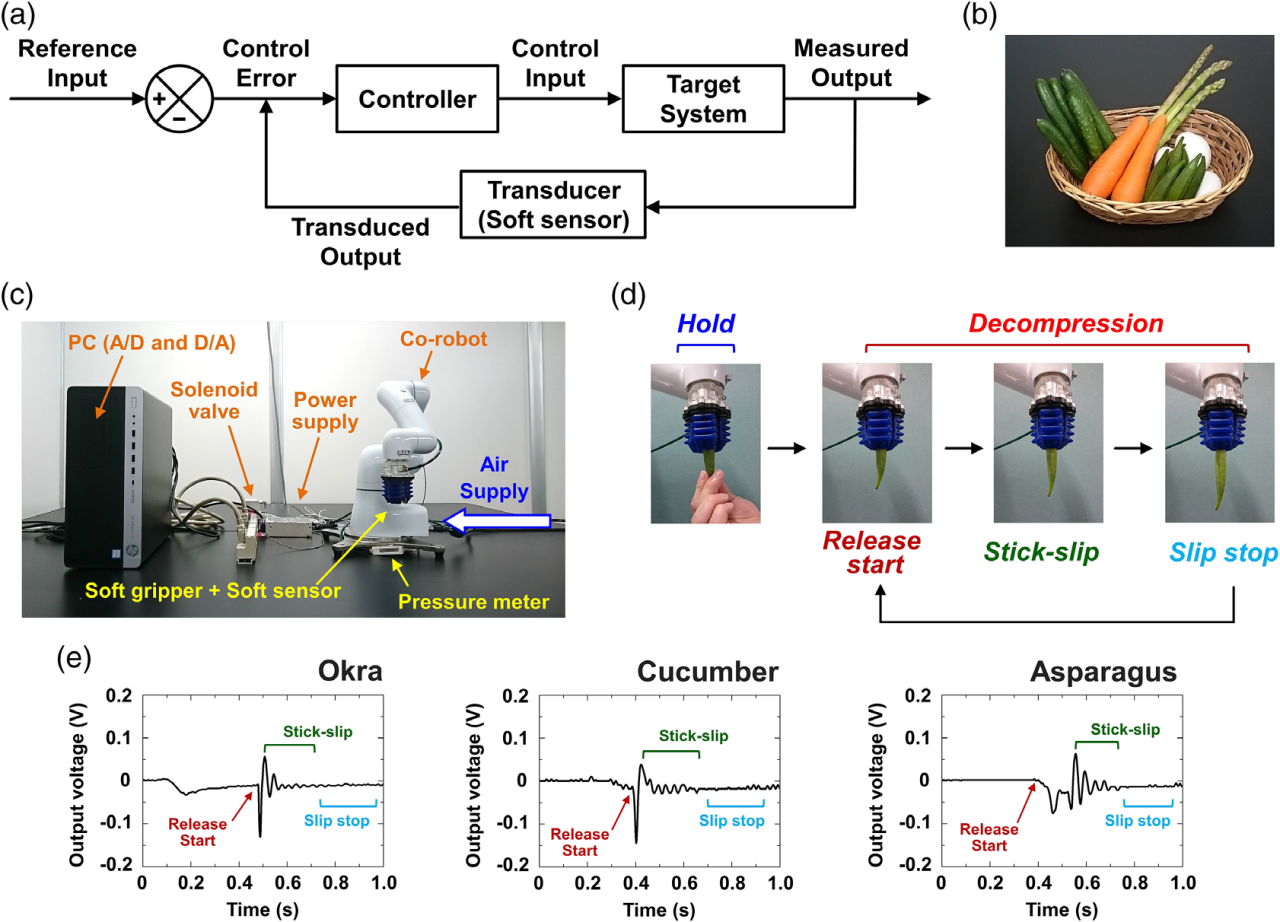
Experiments with the soft sensor mounted on a soft robotic gripper grasping different objects. a) Block diagram of the experimental setup. b) Photographs of fragile greens used as objects to be grasped. c) Experimental setup with our sensor mounted on the gripper and fixed on co‐robot. A/D and D/A converters integrated with PC. Solenoid valve and power supply (24 V) are connected to PC and co‐robot. Solenoid valve controls air compression. d) Photographs of our robotic system with greens during object slippage situations. e) Shear force signals detected using our sensor. Signals consist of hold, slip, and release phases, with their corresponding thresholds indicated as “release start,” “S–S,” and “slip stop,” respectively. Figure S8 and S9, Supporting Information, show additional details.

**Figure** **5** shows the experimental setup and representative experimental results. Figure 5a shows the block diagram of the experimental setup; it consists of a personal computer (PC), digital/analog (D/A) and analog/digital (A/D) converters, air compressor, solenoid valve, 24 V power supply, air pressure gauge, co‐robot, soft gripper, and proposed sensor. In this setup, the detected signal with our sensor and the signal for air supply to the soft hand are synchronized by the D/A and A/D board. When our soft sensor detects slippage signals, the solenoid valve opens and air is automatically supplied to the soft hand. For this reason, it is possible to automatically regrasp objects before they drop down. In this article, as examples of objects to be grasped, we selected fragile greens such as okra, cucumber, and asparagus (Figure 5b). We set up an inflation pressure of 30 kPa for the gripper in consideration of fragile objects. The experimental setup with our sensor mounted on the gripper and fixed on the co‐robot in Figure 5c shows that the air pressure applied to the soft gripper is limited to less than 100 kPa. The simple control process of the feedback system is as follows: 1) the gripper holds the object with an inflation pressure of 30 kPa; 2) air is decompressed at the rate of 1 kPa s^−1^; 3) an analog signal generated by the sensor is sent to the A/D converter; 4) a threshold level is established for the signal converted to digital data using Microsoft Visual Studio programming software; 5) if the intensity of the signal generated by the sensor exceeds the threshold, a command to increase the pressure by 30 kPa is sent through the D/A converter to the solenoid valve; and 6) the soft gripper regrips the object without a training operation.

Figure S8, Supporting Information, shows details of the closed‐loop system. Obviously, we can adjust several properties including the holding pressure and decompression rate. The experiments (see Figure 5d,e) illustrated the high sensitivity of the e‐skin and demonstrated tactile sensing capabilities that allowed the robotic tactile device to interact with easily damageable objects such as fresh okra. By using the shear signals and threshold setting, we produced demonstration videos with and without our original feedback loop system. The soft robot can sense slippage through the simple feedback system by using our soft sensor. Figure S9, Supporting Information, shows experimental results obtained with the soft sensor mounted on a robot gripper for several fragile foods. The evaluated frequency of several S–S signals depending on the objects is shown. Figure S10, Supporting Information, shows the shear force signals detected using sensors with different inflation pressures. By using the soft sensor and feedback system, we produced demonstration videos of the automatic grasping system (Movie S1, Supporting Information). The results indicate a successful experimental demonstration of the high‐sensitivity and high‐speed response of the feedback system for the tactile co‐robot and soft gripper as an e‐skin. In Movie S1, Supporting Information, production of demonstration videos of the automatic grasping system for another object (okra) is shown.

## 3 Conclusions

We fabricated a soft sensor by using a ferroelectric polymer and nanocarbon materials that enable measuring and detecting dynamic shear forces in real time with a high‐speed response. This sensor showed high performance in terms of ferroelectric capabilities and sensing abilities. Our soft sensor is advantageous because when fixed on a co‐robot and soft gripper, it can well grasp fragile objects such as vegetables. We measured the shear force in real time by attaching our soft sensor on a robot gripper. The gripping power of the soft gripper can be controlled in various monitoring tasks as a first step toward integrating its high‐sensitivity directional sensing capabilities, thus demonstrating its potential for various practical soft robotics applications. The feedback system provides a novel control system for operations without the need to train for various tasks, thus demonstrating the potential for novel soft robotics applications such as biomimetic electronic skin.

## 4 Experimental Section

4.1

4.1.1

##### Study Design

In our study, we dissolved all materials used for fabricating the sensor in several solvents and then used them in the printing process shown in Figure 2. Figure S1, Supporting Information, shows details of the printing process. Moreover, in the experiments shown in Figure 3 and 4, the collection rules for all data when applying different ranges of dynamic shear forces were predefined before each experiment. The voltage signal generated from the sensor was detected using an oscilloscope (TDS3054B, Tektronix) with an input impedance of 10 MΩ. As shown in Figure 3, 4, 5, we allowed for the identification of faulty sensor fabrication or bad connectivity at the interfaces. In such cases, the sensor was excluded from the fabrication batch to ensure accuracy.

##### Device Fabrication

The high‐speed response soft sensor was constructed as a condenser device. It was fabricated on a 50 μm thick poly(ethylene naphthalate) (PEN) film (Q65HA, DuPont) and fixed to a glass carrier. A cross‐linkable poly(4‐vinyl‐phenol) (PVP) (436224, Sigma‐Aldrich) solution consisting of a mixture of PVP and melamine resin (418560, Sigma‐Aldrich) with 1‐methoxy‐2‐propyl acetate (Kanto Chemicals 01948‐00) as the solvent was spin coated onto the PEN film as the planarization layer. Next, to orient the crystallinity of the ferroelectric layer, PEDOT:PSS (Clevios SV4 STAB, Heraeus) was mixed with a GO (G0443, Tokyo Chem. Industry) and NMP (872‐50‐4, Tokyo Chem. Industry) with 1.0 wt% concentration. After the aforementioned materials were mixed, they were agitated by a rotation and revolution mixer (AR‐100, Thinky) for 10 min. The solution was formed on the planarization layer by screen printing (MT320T, Micro‐Tec.) to form the bottom electrode. The electrode was annealed at 150 °C for 30 min, and it had a thickness of ≈500 nm. Furthermore, a 2000 nm thick P(VDF–TrFE) layer (62‐010, Piezotech, VDF:TrFE molar ratio of 75:25) with a carboxylic acid functionalized SWCNT (652490, Merck) [0.075 wt% for P(VDF–TrFE)] was formed by screen printing and annealing at 135 °C for 1 h. Before printing, this solution was filtered using a membrane filter with a pore size of 1 μm. Figure S11, Supporting Information, shows details of the solution preparation. In the aforementioned materials system, the solution's concentration was 12 wt% and its viscosity was ≈800 mPa s. Moreover, the top electrode of the sensor was formed with the same materials as the bottom electrode by using a screen printer and by annealing at 135 °C for 30 min. Figure S4, Supporting Information, shows the materials systems used in this article. Next, a 200 nm thick polyparaxylene layer (diX‐SR, KISCO) was thermally deposited as a 300 nm thick passivation layer. Finally, a 50 μm thick PI layer (5434 3M) (PI) as a fractionable layer was attached on the outermost surface of the sensor, as shown in Figure 1a,b. The coefficient of dynamic friction of the PI film was 0.1; the actual measurement value is shown in Figure S3, Supporting Information.

##### Soft Robot Platform Setup

The sensor was fixed by a PI film on a soft gripper (SRT gripper, SFG‐FTN3‐M3028, IDEC Factory Solutions) mounted on a co‐robot arm (COBBOTA, Denso Wave). The operation language of the co‐robot used in this article was PAC SCRIPT. The co‐robot was programmed to perform a series of predefined movements, as defined by an experimental protocol with a digital controller that could perform a pick‐and‐place operation depending on the signal generated by the sensor. The closed‐loop system for robotic control considered the sensor signal through the A/D and D/A convertors as the input and controlled the gripper force when the signal reached a predefined output voltage threshold. A controllable software interface developed using Microsoft Visual Studio was used for communication between the sensor and the computer controlling the gripper using compressed air with a pressure of 30–60 kPa. The system was implemented in the C programming language.

## Conflict of Interest

The authors declare no conflict of interest.

## Data Availability Statement

Research data are not shared.

## Supporting information

Supplementary Material

Supplementary Material

Supplementary Material
