# Supplementary material for: Artificial Cutaneous Sensing of Object Slippage using Soft Robotics with Closed‐Loop Feedback Process
Source: Small Sci. 2021 Feb 7;1(3):2100002. doi: 10.1002/smsc.202100002 (PMC11935906; doi:10.1002/smsc.202100002)
Supplement: Supplementary file 1 — Supplementary Material [file SMSC-1-2100002-s001.docx]

Supporting Information

Artificial Cutaneous Sensing of Object Slippage using Soft Robotics with Closed-Loop Feedback Process

Tomohito Sekine,* Yi-Fei Wang, Jinseo Hong, Yasunori Takeda, Reo Miura, Yushi Watanabe, Mai Abe, Yoshiki Mori, Zhongkui Wang, Daisuke Kumaki, Fabrice Domingues Dos Santos, Atsushi Miyabo, Sadao Kawamura, Shizuo Tokito*


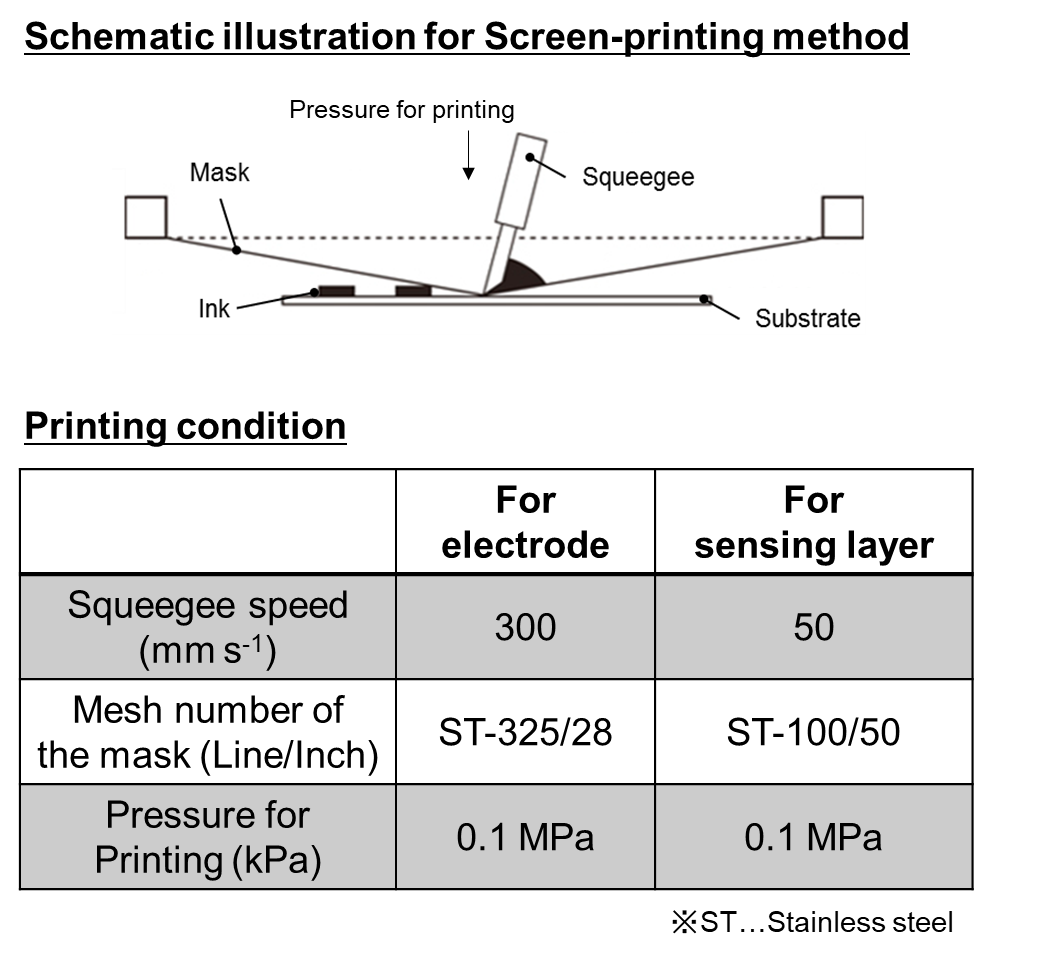


**Figure S1 Schematic illustration and screen-printing of fabricated sensor.** The printing process involved ink, a mask, a squeegee, and the substrate. The table below lists the printing conditions.


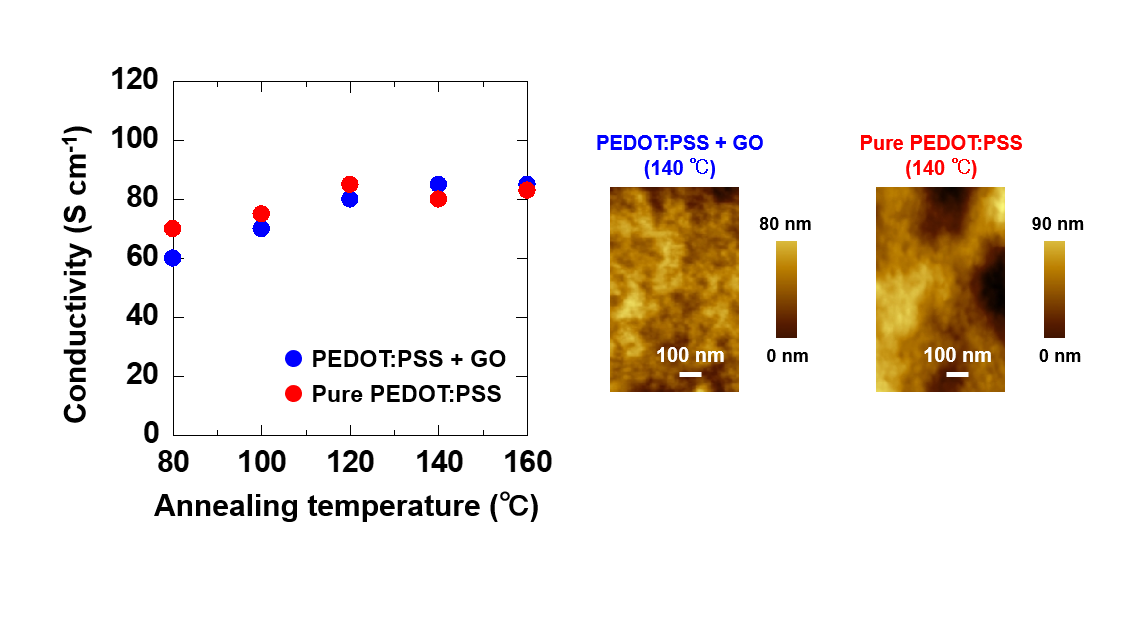


**Figure S2** **Conductivity changes in electrode as a function of annealing temperature.** The electrode was made of PEDOT:PSS and a GO. AFM images on the right-hand side show the surface morphology of the electrode with and without GO annealed at 140 °C.


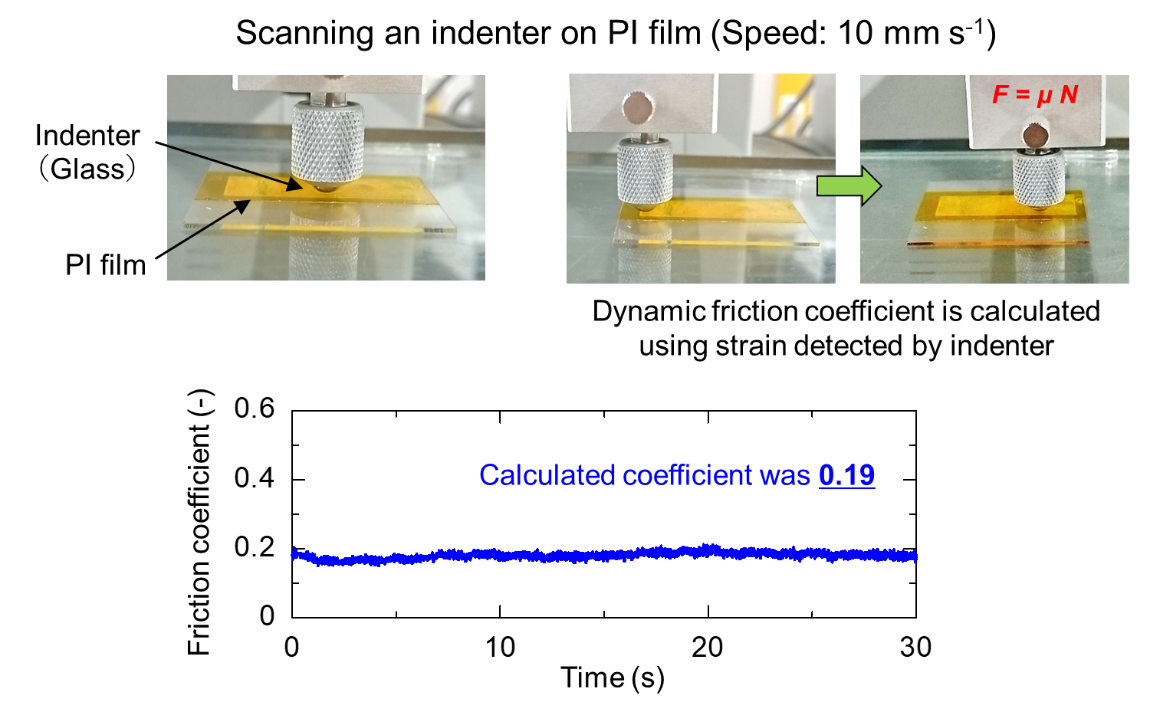


**Figure S3** **Measurement of dynamic friction coefficient of our sensor**. The sensor surface was formed by a PI film. The scanning speed of the indenter on the PI film was 10 mm s^-1^. The calculated dynamic friction coefficient was 0.19. The Haptics & Material Characterization facility (TL201Tt, Trinity-Lab.) was used for measuring the coefficient.


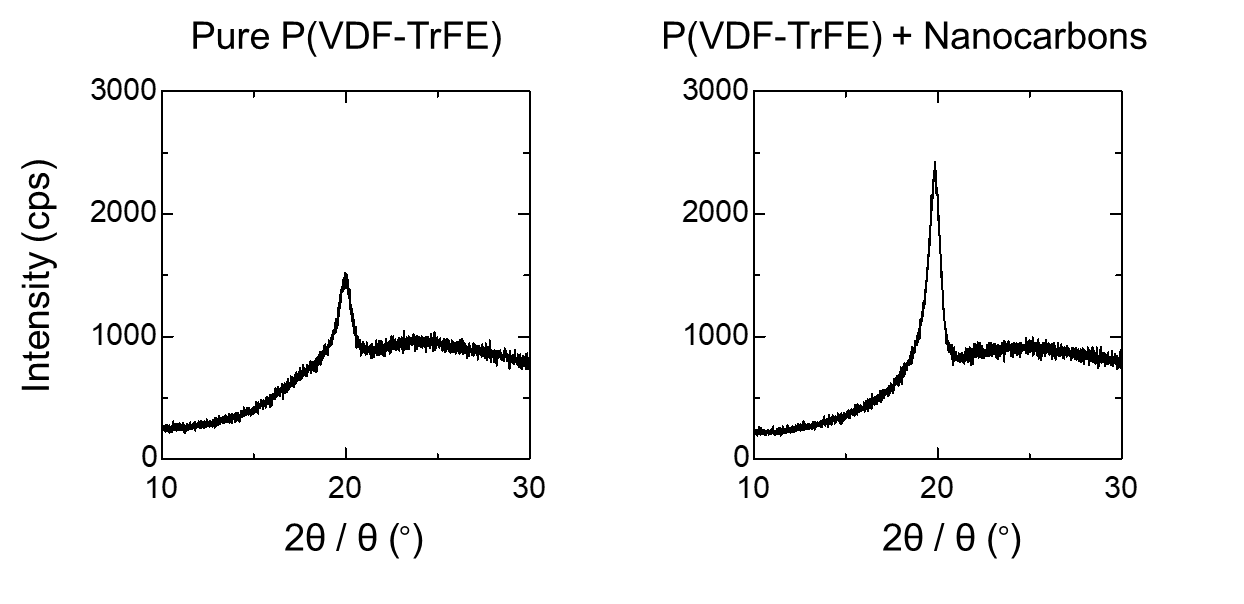


**Figure S4 Measured XRD spectrums for pure P(VDF-TrFE) and P(VDF-TrFE) + SWCNT.**


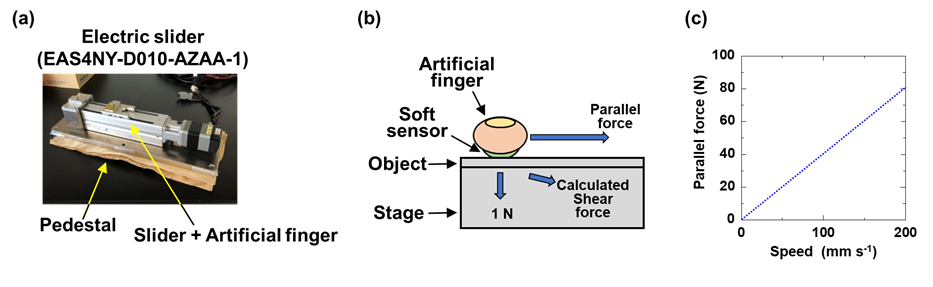


**Figure S5** **Setup for shear force application with sensor.** (a) Overview of our shear force application system. (b) Cross-sectional image of this system. The sensor was attached on an artificial finger that was fitted on the slider. For generation of shear force, we applied 1.0 N in the perpendicular direction and several parallel directions. The perpendicular force of 1 N is adjusted by the height of the artificial finger for the stage. (c) Relationship between moving speed of the artificial finger for parallel direction and force.


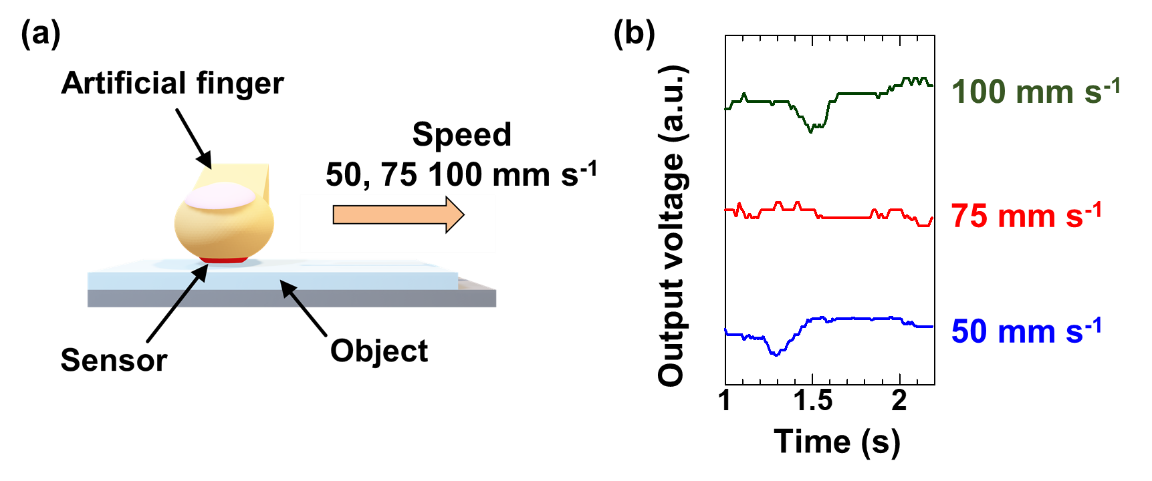


**Figure S6** S**ensor response upon applying a shear force.** (a) A schematic 3D illustration of the measurement setup for characterizing the sensing ability with difference share speeds. The speeds were 50, 75, and 100 mm s^-1^. (b) A periodic signal generation when the shear force was applied. The shape of signals depended on applied speeds.


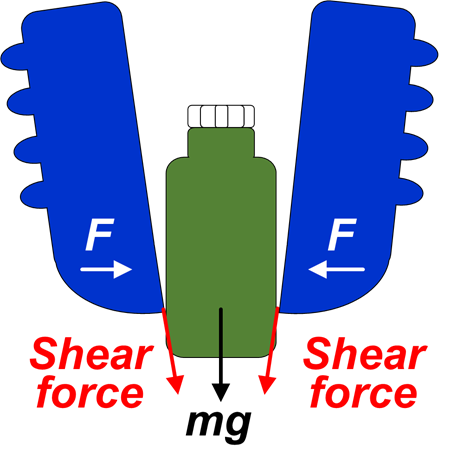


**Figure S7 Cross-sectional simple model describing shear force generation in falling direction.** The force included the handling force and gravity acting on the object.


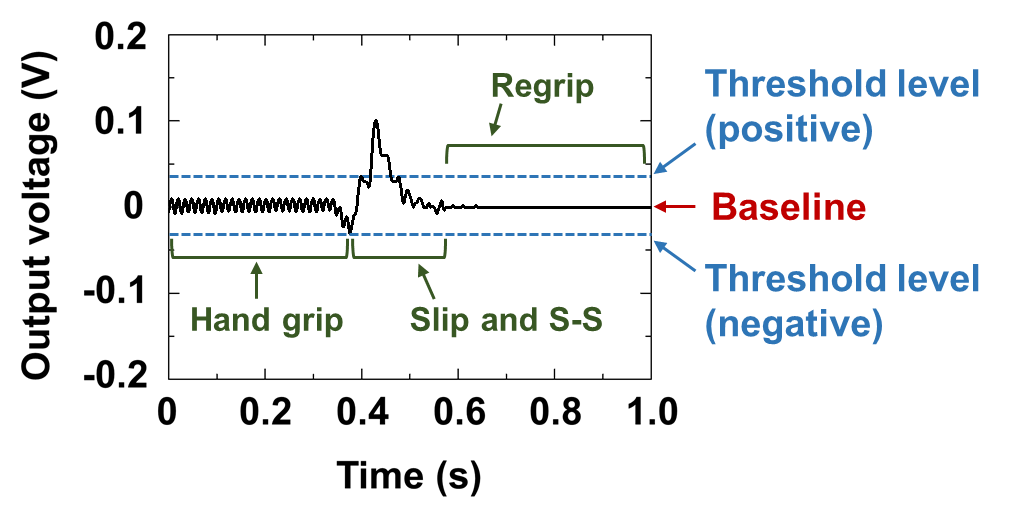


**Figure S8 Description of feedback system using sensor.** We set the threshold levels for the detected signal in both the positive and the negative potentials. When the output voltage exceeded the threshold levels, a signal commanding regripping was transmitted to the hand.


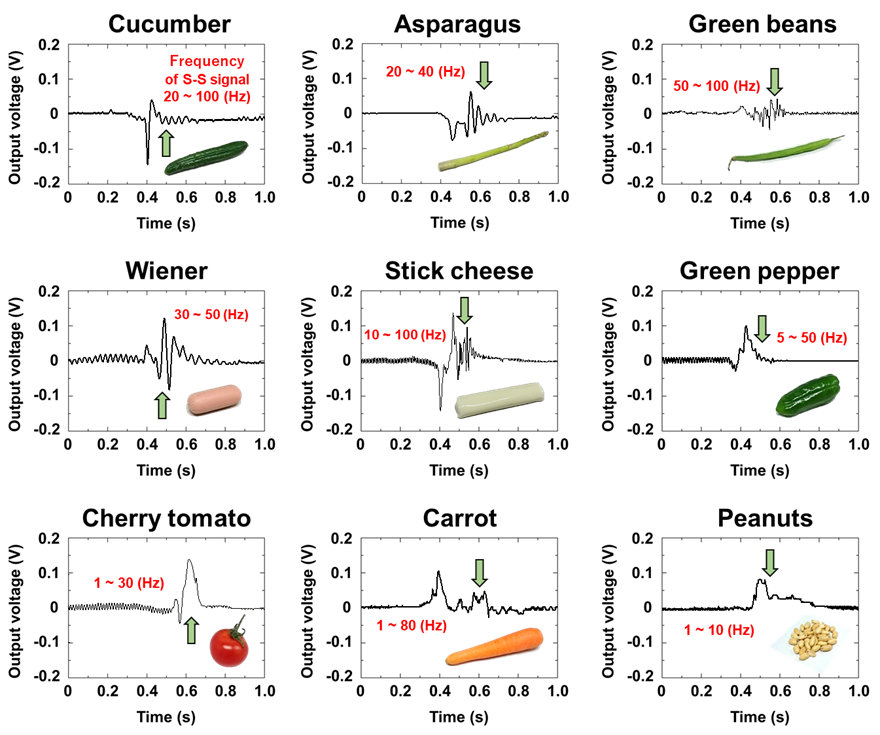
**Figure S9 Experiments with soft sensor mounted on a robot gripper for several fragile foods.** Shear force signals detected using our sensor. The signals consist of hold, slip, and release phases. The inset arrow indicates the S-S signal, and the evaluated frequency of several S-S signals is displayed as a graph.


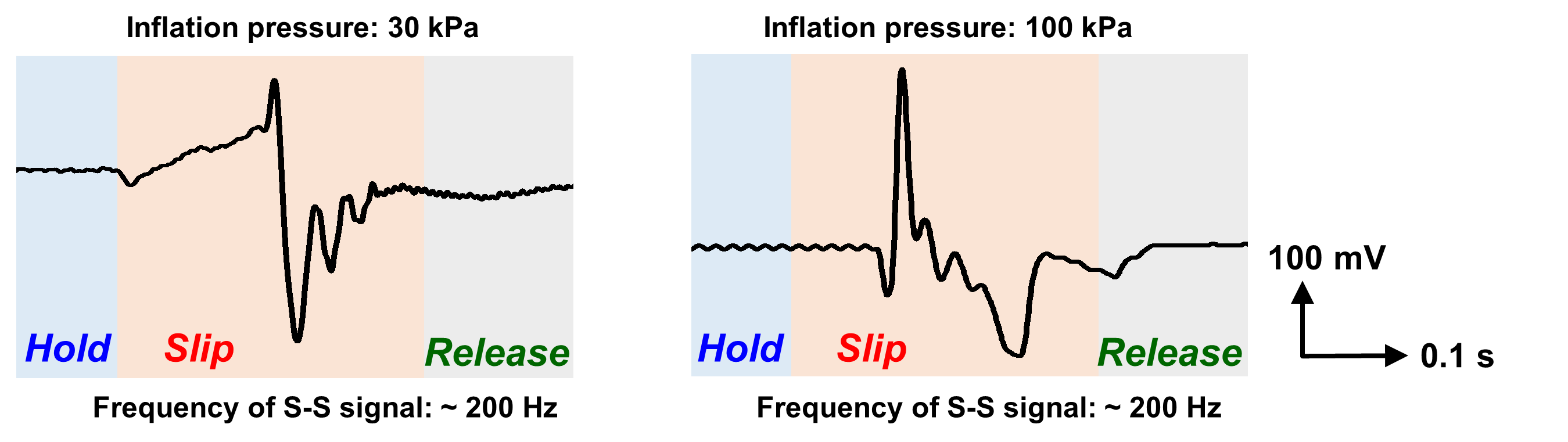


**Figure S10 Shear force signals detected using sensors with different inflation pressures.** A glass bottle was used as the object to be grasped. The generated voltage levels are the same with both pressures. Although the shape of signals differed, both evaluated frequencies of the S-S signal are under 200 Hz.


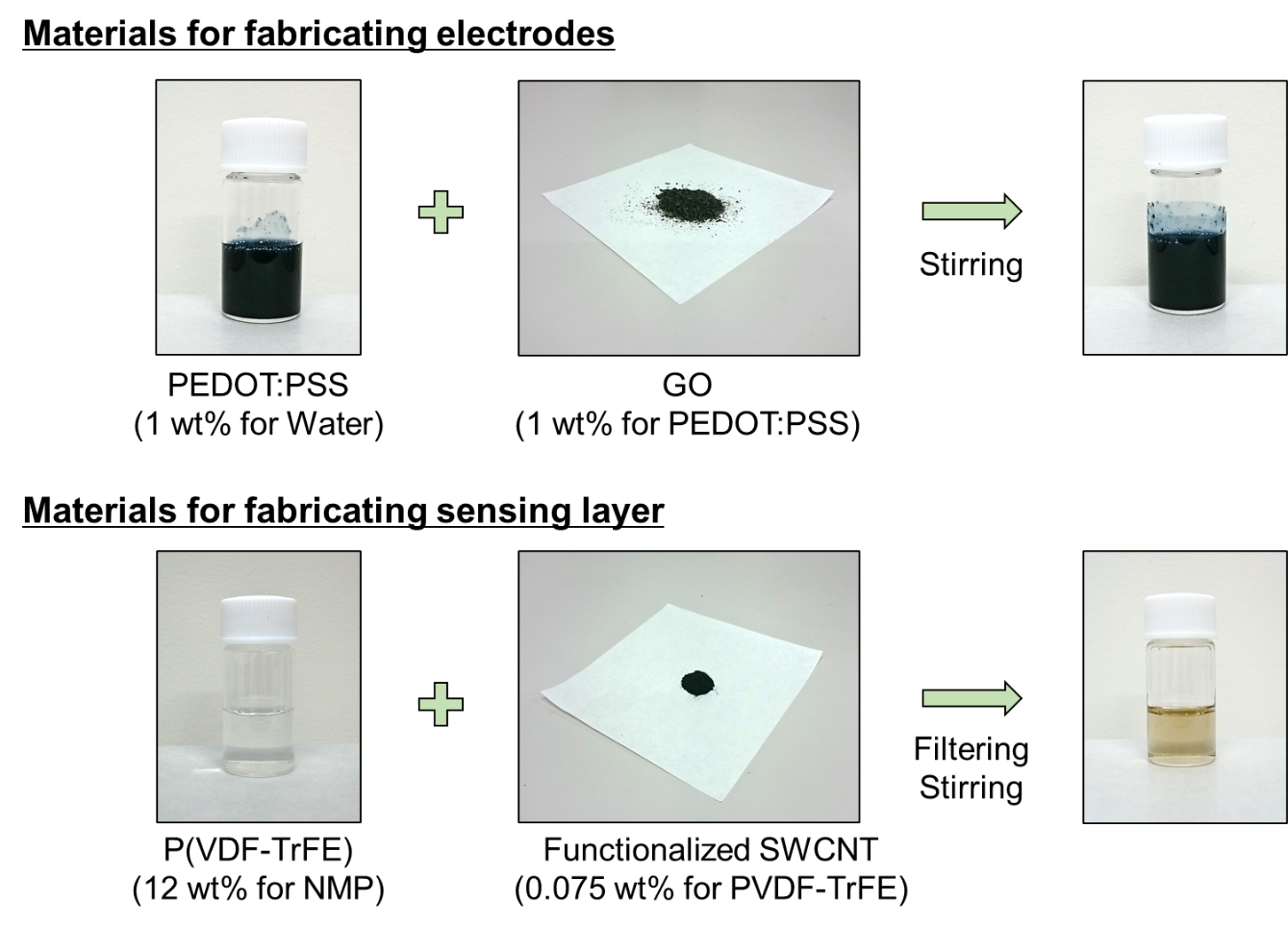


**Figure S11 Material system for fabricating electrode and sensing layer**. The electrodes were fabricated using a solution of PEDOT:PSS (1 wt% for water) and a GO (1 wt% for PEDOT:PSS solution). GO was added to PEDOT:PSS and the solution was stirred for 10 min. Next, for the sensing layer material, SWCNT was added to the P(VDF-TrFE) solution and the solution was stirred for over 24 h. Finally, we filtered the solution and left it standing for degassing.


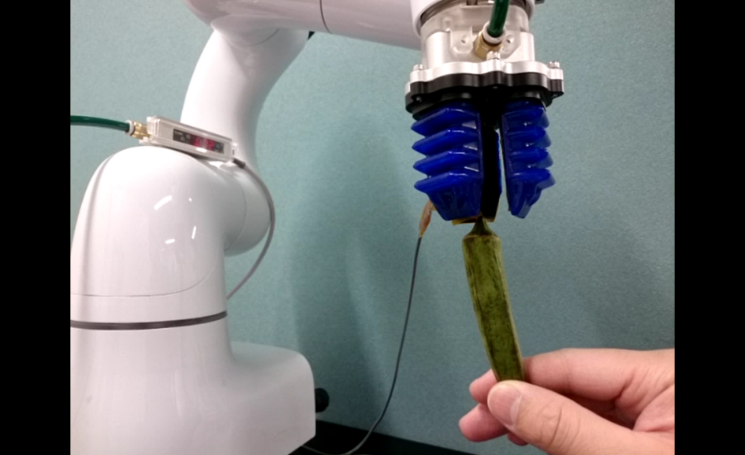


**Movie S1 production of demonstration videos of the automatic grasping system for another object (Okra).**
